# Estimating the density of small mammals using the selfie trap is an effective camera trapping method

**DOI:** 10.1007/s13364-022-00643-5

**Published:** 2022-07-22

**Authors:** Ana Gracanin, Todd E. Minchinton, Katarina M. Mikac

**Affiliations:** Centre for Sustainable Ecosystem Solutions, School of Earth, Atmospheric and Life Sciences, Faculty of Science, Medicine and Health, University of Wollongong Australia, New South Wales, Australia

**Keywords:** Abundance, Camera trapping, Capture probability, Capture-mark-recapture, Density, Live trapping, Monitoring, Small mammal, Trap comparison

## Abstract

Camera trapping to study wildlife allows for data collection, without the need to capture animals. Traditionally, camera traps have been used to target larger terrestrial mammal species, though recently novel methods and adjustments in procedures have meant camera traps can be used to study small mammals. The selfie trap (a camera trapping method) may present robust sampling and ecological study of small mammals. This study aimed to evaluate the selfie trap method in terms of its ability to detect species and estimate population density. To address this aim, standard small mammal live trapping was undertaken, immediately followed by camera trapping using the selfie trap. Both methods were set to target the arboreal sugar glider (*Petaurus breviceps*) and semi-arboreal brown antechinus (*Antechinus stuartii).* The more ground-dwelling bush rat (*Rattus fuscipes)* was also live trapped and recorded on camera. Across four survey areas, the probability of detection for each of the three species was higher for selfie traps than for live trapping. Spatially explicit capture-recapture models showed that selfie traps were superior at estimating density for brown antechinus and sugar gliders, when compared to simulated live trapping data. Hit rates (number of videos per various time intervals) were correlated with abundance. When correlating various hit rate intervals with abundance, the use of 10-min hit rate was best for predicting sugar glider abundance (R^2^ = 0.94). The abundance of brown antechinus was estimated from selfie traps using a 24-h hit rate as a predictor (R^2^ = 0.85). For sugar gliders, the selfie trap can replace live trapping as individuals can be identified through their unique facial stripes and natural ear scars, and thus used in capture-recapture analysis. This method may be useful for monitoring the abundance of other small mammal species that can also be individually recognized from photographs.

**Supplementary Information:**

The online version contains supplementary material available at 10.1007/s13364-022-00643-5.

## Introduction

Motion sensing camera traps have become an important method in ecological study and surveys for a wide range of animal species. Camera traps have been used to study distribution (e.g., Rovero et al. 2013), abundance (e.g., Silver et al. 2004), behavior (e.g., Šprem et al. 2015; Gracanin et al. 2019) and community structure (e.g., Martin-Albarracin et al. 2015). These devices facilitate large-scale spatial and temporal data collection (e.g., Swanson et al. 2015). This has led to the development of Artificial Intelligence-based platforms for processing resultant large datasets (e.g., Norouzzadeh et al. 2018), and their associated software packages (Young et al. 2018).

Camera trapping is often highly effective and indeed sometimes necessary when studying rare and endangered species (Pérez et al. 2011; Bezerra et al. 2014; McDonald et al. 2015). Removing the need to directly observe or physically handle an animal has meant that monitoring them can be achieved with minimal negative impacts on the target species or populations. Traditionally, camera traps have been used to target larger terrestrial mammal species, although recently novel methods and adjustments in procedures have meant camera traps can be used to study reptiles (Welbourne 2013; Hobbs and Brehme 2017), arboreal species (Gracanin et al., 2018, 2020; Gregory et al., 2014; Whitworth et al., 2016) and small mammal species (De Bondi et al. 2010; McCleery et al. 2014; Villette et al. 2016, 2017; Mos and Hofmeester 2020; Thomas et al. 2020; Littlewood et al. 2021).

Small mammals are susceptible to the effects of habitat fragmentation, and monitoring their diversity, distribution and population dynamics is important for their conservation (Andrews 1990; Gaines et al. 1992; Andren 1994; Gelling et al. 2007). Camera trapping allows for the study of small mammals over large spatial and temporal scales, in difficult to access locations, with reduced field-based work and animal welfare impacts. This highlights the importance and great potential that camera trapping has in studying small mammals (Costa et al. 2005; Bumrungsri et al. 2013). Despite the vast amounts of data that cameras can potentially collect, there has been relatively little application of such methods in small mammal species (Di Cerbo and Biancardi 2013; Rovero et al. 2013; Shadbolt 2014; McDonald et al. 2015; Yamada et al. 2016; White et al. 2017; Gracanin et al. 2019) compared to larger mammal species (see review by: Mccallum, 2013). More recently, a review into camera trapping studies on the African continent identified small mammals as an underrepresented taxa within the camera trapping studies reviewed (Agha et al. 2018).

Live trapping is the standard method for estimating population density of small mammals, with aspects such as trap detection probability and survey lengths (Batzli 1992; Prevedello et al. 2013; Gentile et al. 2018; Fuentes-Montemayor et al. 2020), and mark retention and recognition (Jung et al. 2020), as important considerations. Estimating densities with camera traps is achievable when the focal species features markings that allow for individual identification (e.g., Karanth 1995) or when animals have been caught and marked (Jung et al. 2020), so that mark-recapture models can be used to estimate densities. However, where individuals cannot be distinguished, then hit rate data (number of camera trap events) can be applied to random encounter models (REMs) to estimate densities (Rowcliffe et al. 2008). The assumptions of REMs are not always met in camera trapping studies (e.g., bait is used to attract elusive species); therefore, calibrating hit rates with density estimates obtained through another method (e.g., live trapping) is one way to approach this (Villette et al. 2016, 2017). This calibration method been successfully used to estimate population densities of small mammal species, such as red-backed voles (*Myodes rutilus*) and deer mice (*Peromyscus maniculatus*) (Villette et al. 2016), and red squirrels (*Tamiasciurus hudsonicus*) (Villette et al. 2017).

In the context of small mammals, cameras have limited focus ranges resulting in low-resolution images that can then lead to false-positive identification of species (Meek et al. 2013). Some have modified camera placement, for example mounting cameras to face down, parallel with the ground, to obtain images in higher clarity (De Bondi et al. 2010; Gray et al. 2017; Dundas et al. 2019) or placed in protective housing (McCleery et al. 2014; Mos and Hofmeester 2020). More recently, purpose-designed camera traps have been made to obtain facial images of small mammal species, a method aptly named the selfie trap (Gracanin et al. 2018). The selfie trap consists of a close focus lens placed on a trail camera, with a bait holder of a known size affixed in front of the camera, all housed in a PVC pipe. When small mammals enter the selfie trap, they are attracted to the bait and typically face the bait holder head on which facilitates the capture of an image of the animal's face. Resultant images are often in sharp focus at high resolution, which facilitates identification of species of a similar appearance, that would otherwise be indistinguishable using standard camera trapping methods (e.g., Meek and Vernes 2015). However, the selfie trap method has yet to be evaluated for its ability to estimate occupancy, abundance or density of small mammal populations (Gracanin et al. 2018). Therefore, this study aimed to test the effectiveness of selfie traps, primarily targeting the arboreal, sugar glider (*Petaurus breviceps*) in addition to the semi-arboreal (*Antechinus stuartii*). Enough captures were obtained for the more ground-dwelling bush rat (*Rattus fuscipes*) to also include in the analyses. Specifically, we tested the following questions:

Q1. Is the probability of detection of small mammals higher for selfie traps when compared to live trapping? We predicted that selfie traps have greater detection probabilities as cameras are an “open” trap throughout the night which would allow for greater efficiency of detecting multiple species.

Q2. Does the duration and trigger interval in selfie trap surveys influence detection rates of individuals and species? Kays et al. (2020) found that a two-week survey period was most efficient but recommended 3–4 weeks to increase precision. We predicted that selfie trap survey periods of one, two, three and four weeks will impact the number of individuals observed on camera. We predicted that different trigger intervals (time delay between when the motion sensor is activated to record) will also affect the number of individuals observed.

Q3. Can small mammal abundance be estimated using capture rates from selfie traps? We predicted that the amount of selfie trap footage will correlate with estimated small mammal abundance, and that different hit rates of footage (number of videos per various time intervals) will affect this correlation, as per Villette et al. (2016, 2017).

Q4. Can small mammals be individually identified using the selfie trap in order to estimate density at a site? We predicted that the data collected from selfie traps will be able to accurately estimate single-season density of the target species, the sugar glider, compared to a simulated live-trapping dataset.

Q5. Is camera trapping using the selfie trap a more cost-effective method than standard live trapping? We predicted that using the selfie trap under this study’s conditions would be more cost-effective over the long term.

## Materials and methods

### Study site

**Fig. 1:**
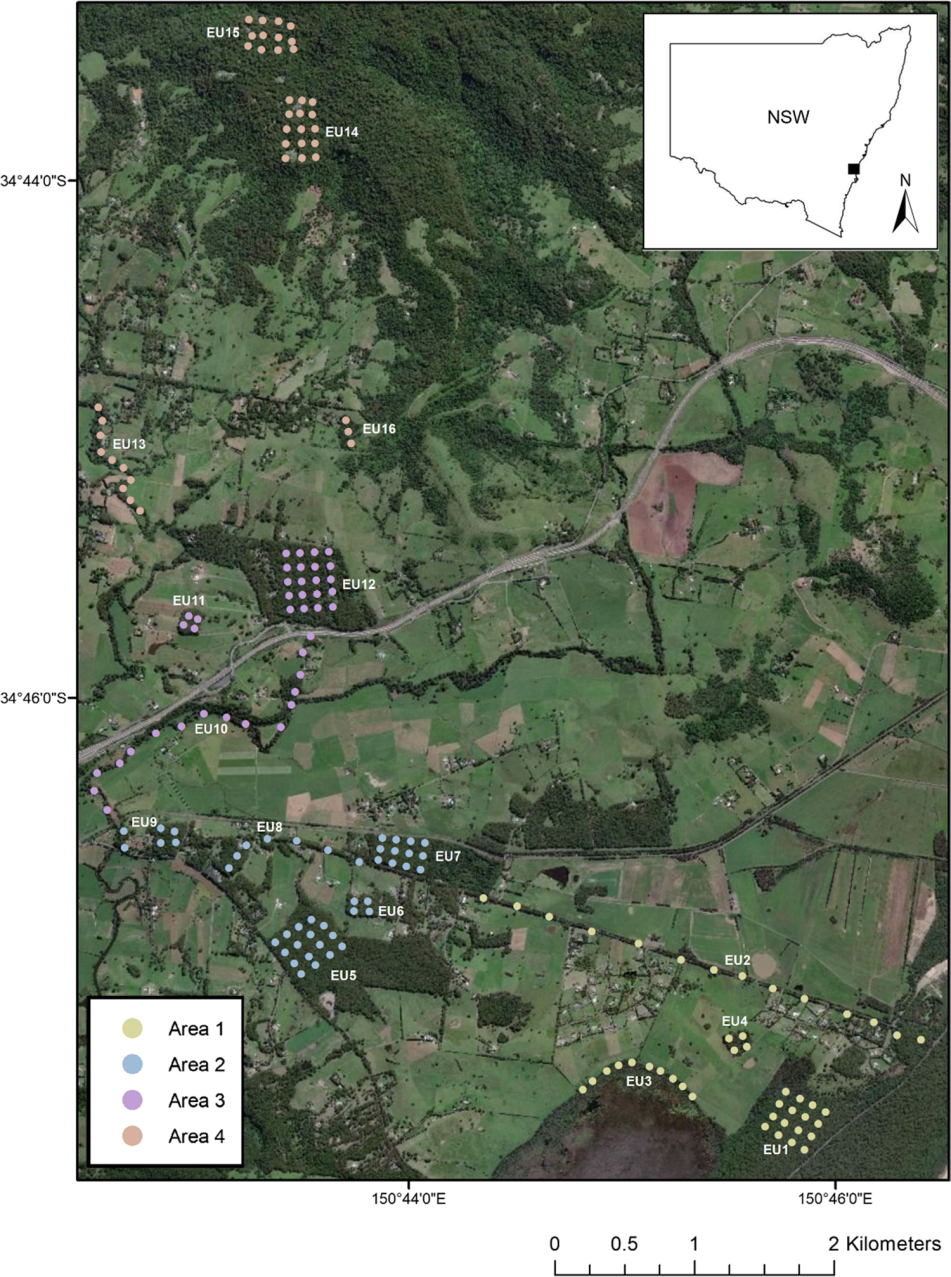
Trap locations in the study landscape surrounding Berry, NSW (*n* = 164), where each live trap (7 nights) shown on the map was immediately replaced with a selfie trap (28 nights). Areas one to four were surveyed immediately after each other (August to November 2019). Distinct trapping grids and transects are labelled as experimental units (EU). The number of traps in each area was: Area 1 = 44, Area 2 = 45, Area 3 = 40, and Area 4 = 40. Imagery base map is from NSW Government © Spatial Services 2021

This study was conducted in a fragmented landscape surrounding the township of Berry, New South Wales, Australia, 111 km south of Sydney (Fig. 1). Sites (*n* = 164) were chosen to occur within the project area of a planned wildlife corridor (Berry Bush Links; Great Eastern Ranges initiative) (GER 2021). Most of the landscape is gentle to undulating, with floodplains occurring around the township of Berry. The geology mainly comprises of Volcanic, Siltstone and Alluvial deposits (Rose 1966). Vegetation east of the study area at Seven Mile Beach National Park (16 m above sea level) was a combination of littoral rainforest and blackbutt-dominated (*Eucalyptus pilularis*) dry sclerophyll forest (Gracanin et al. 2019). Near this park is Coomonderry Swamp, a large semi-permanent freshwater wetland with the dominant tree species being *Casuarina sp.* Majority of the landscape was fragments of either blackbutt-dominated or turpentine-dominated (*Syncarpia glomulifera*) open and closed forests, with creek lines mostly containing *Casuarina sp.* Sites furthest to the west on the Illawarra Escarpment (410 m above sea level) contained sub-tropical rainforest. At the closest weather station, Kiama Bombo Headland, annual rainfall was 646.0 mm and mean monthly temperatures ranged between 25.4ºC (February) and 10.1ºC (July) (Australian Bureau of Meteorology 2021).

### Live trapping

Across the landscape, 164 live traps were set up in total and these were divided into four survey areas that targeted key areas of the Berry corridor (Fig. 1). Each area survey was performed four weeks apart, with area one live trapping starting on the 13 Aug 2019, before sequentially moving onto the next area. Each area was surveyed in distinct linear transects, or grids, which was dependent on the fragment of habitat being surveyed (Fig. 1). These formed 16 “experimental units” (Fig. 1). Traps were spaced 100 m apart in grids (Quin et al. 1992), and an average of 250 m along linear transects (dependent on private property limitations).

**Fig. 2:**
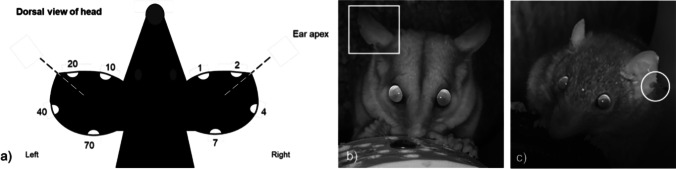
For small mammals captured, each individual was given a unique code within each area that corresponded with ear notching. The code combinations of this is shown in (**a**). The appearance of these unique ear codes on selfie trap footage is shown in two examples: **b**) a female sugar glider (*Petaurus breviceps*) is shown with the code “03” applied (ear punch 1 and 2); and **c**) a female antechinus (*Antechinus stuartii*) is shown with code “40” applied (ear punch 40)

Elliott A traps (Tasker and Dickman 2001) were secured onto wooden platforms (drilled onto trees), at heights of 2 m above ground to target arboreal sugar gliders. The entrance to traps faced the tree, and peanut butter, honey and oats were used as bait (Suckling and Macfarlane 1983; Campbell et al. 2018). A honey water mixture was sprayed up and down the tree (3-5 m) that the trap was secured to, as well as surrounding foliage (Jackson 2000). Traps were checked for seven mornings before they were packed down (seven nights was the maximum length of a trapping session as allowed by the animal ethics protocol). Animals caught had a small genetic sample taken; a 2 mm clipping on the ear margin (Nowack et al. 2015; Knipler et al. 2021). Each individual animal was given an unique code based on the position of this clipping (Fig. 2). This allowed for recaptures of individuals to be recorded through both live trapping, and on camera footage, later collected through selfie traps.

### Camera trapping using the selfie trap

**Fig. 3:**
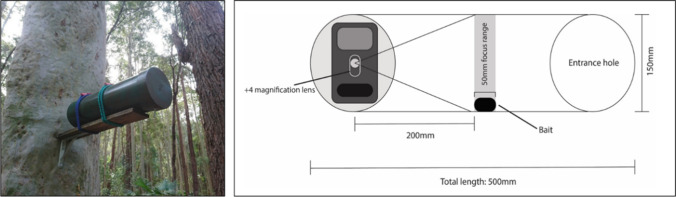
Example of a selfie trap positioned on a platform, 2 m off the ground to target arboreal species. Inside the selfie trap is a camera with an altered focal distance for close recording of small mammal species

Following the last morning of live trapping at each area, a selfie trap (Fig. 3) was placed on the same platform as the Elliott trap and left to record for four weeks (*N* = 164 selfie traps). The selfie trap has a plastic bait holder with small holes for limited bait (peanut butter, honey and oats) access. This bait was positioned within the modified focal range of a Browning Recon Force 4K (BTC-7-4 K) camera (Gracanin et al. 2018). Selfie traps were rebaited and resprayed with honey water 14 days into the 28-day survey blocks.

The cameras were set to record a 20 s, high definition (60 FPS) video clip. The interval trigger was set to 1 min for area one, and five mins for area two, but later changed to 10 min for the other areas due to the large dataset collected from the shorter intervals (30,423 videos were recorded at area one and 24,213 videos were recorded at area two).

### Camera data recording

Individual video files were allocated unique identifiers based on site and temporal sequence using the software *Advanced Renamer* (Jensen 2020)*.* Video files containing footage of various species were allocated to species specific folders created for each camera. Within these species folders, individuals were identified by either unique pelage patterns, scars, or ear notches from live trapping. Profiles were created for each individual to aid in identification, and this was performed by the one observer. A strict procedure was developed to ensure only distinguishable animals could be identified as an individual, and this is outlined in the Supplementary Material. Furthermore, a subset of the data was analyzed by three other observers to confirm the accuracy of the procedure (Supplementary Material).

The software *BulkFileChanger* (Sofer 2021) was then used to collect the date and time for every video file in bulk, for every folder. Using the common lowest interval setting placed on cameras across all areas, only footage recorded ten minutes after the previous trigger was used for the areas one and two datasets. This was calculated using the assess temporal independence function in the R package *camtrapR* (Niedballa et al. 2016) in R Studio (RStudio Team 2020).

### Probability of detection

Occupancy modeling for live trapping and camera trapping of antechinus, bush rats, and sugar gliders was performed (R package "unmarked"; Fiske and Chandler 2011). Single-season occupancy models were used to estimate the probability of detecting each species at each site (MacKenzie et al. 2002). Modeling for both live trapping and camera trapping (using the first seven nights of camera trapping) was conducted based on a constant occupancy rate *p*(.). As we were strictly interested in how the two survey methods operated when placed in completely random locations, we did not use covariates (De Bondi et al. 2010). Analyses were performed per area, to separate the influence of season on detection probabilities.

### Selfie trap survey durations

To determine the effect of various selfie trap survey durations on detecting individuals within species, the dataset was divided to so that the number of individuals identified on camera was compared among 7-, 14-, 21- and 28-day datasets.

### The effect of trigger delay

The effect of sampling frequency at the individual camera level was then tested using the dataset from area one. A high number of recordings at area one provided a unique opportunity to compare the effect of trigger interval as the dataset was collected with the camera set to a one-minute delay. We explored the effect of 1-, 2-, 3-, 4-, 5-, 10-, 15-, 20-, 30-, 60-, 120-, 240-, 480-, 720- and 1440-min delays. This was achieved by using the assess temporal independence function in the R package *camtrapR* (Niedballa et al. 2016)*.* This meant that at each site, after the first video file was recorded, the selected interval time had to pass before the next video was to be included in the dataset. The number of individuals for each species was then plotted against each interval dataset.

### Camera hit rate analysis

A linear regression of the camera hit rate (number of videos) per day on abundance (number of distinct individuals observed) was tested, using data pooled at each experimental unit (EU) (Fig. 1). Hit rates were calculated for each experimental unit as the total number of videos of the species recorded within specified time windows, divided by 28 nights, to calculate a daily hit-rate average (Villette et al. 2017). The hit rate intervals tested were 10, 30, 60, 90, 120, 150, 180, 210, 240, 720, 1440, 2160 and 2880 min. Linear regressions were used to determine if each hit rate could predict estimated abundance for sugar glider and brown antechinus.

### Density analysis

Using the R package *secr* (Efford 2015), three datasets were used to compare the effectiveness of selfie traps to estimate population density: 1) live trapping; 2) ear marked individuals on camera; and 3) all unique individuals identified on camera (both ear marked and those uniquely identified from cameras alone). Additionally, the Peterson method for single marking event (live trapping) and single recapture event (camera trapping) was conducted using the estimator from Bailey (1952).

The seven nights of live trapping did not yield enough recaptures to warrant analysis through spatially explicit capture-recapture models (nor was the study able to be repeated as planned the following year due to COVID-19 restrictions). Instead, the four-week camera dataset was converted to simulate a live trapping dataset. This assumed that the first visitor to the trap was “live captured,” and thus the trap was closed, and no other observations recorded for the remainder of the night. However, as selfie traps were positioned to target the arboreal sugar glider, this affected the capture of ground-dwelling brown antechinus and bush rats. Thus, to increase the number of observations for these more ground-dwelling species, the first individual recorded for that species each night was recorded for each simulated live trapping dataset. These live trapping datasets therefore reflect a higher detectability than otherwise anticipated. In addition to this, we acknowledge that selfie traps have a greater detectability than live traps due to individual return rates likely being higher as there is no negative capture experience to affect individual behavior (Stryjek et al. 2019).

For the three datasets (live-trapping simulated, individuals with ear markings on camera, and all individuals with ear markings and unique features on camera) at each area, we performed spatially explicit capture-recapture (SECR) analysis. The detection matrix used was based on whether an individual was detected each night. Populations were assumed closed during the sampling period. For all SECR analyses, a habitat buffer was selected using the “suggest.buffer” function in *secr*, with woody vegetation canopy cover (Office of Environment and Heritage 2016) used to mask available habitat for density estimations.

Firstly, we compared 15 a priori SECR models of each species density, for each dataset. The models assumed animals were distributed following a homogenous Poisson process. These models included possible factors affecting density (D), such as the scale of movement (sigma), and the probability of detection (g0), and different detection functions for half-normal, exponential and hazard-rate. We evaluated models with Akaike’s Information Criterion corrected for small sample size (AICc), and results of the parsimonious model were chosen for comparison with the other estimation techniques. For all analyses, a null model using the hazard-rate function performed best.

### Cost analysis

A cost analysis was conducted using the conditions of this study. In our study, two teams (each led by an experienced animal handler; the remainder volunteers) and average times taken to conduct fieldwork, were used. Time taken included travel time to and between sites. Time taken to sort footage was assumed to be conducted by an already experienced and trained individual, for a dataset collected under a 10-min interval. University casual employment pay rates were used, and average usage of consumables were used to calculate costs. All expenses are presented in Australian dollars.

## Results

### Is the probability of detection of small mammals higher for selfie traps when compared to live trapping?

**Fig. 4:**
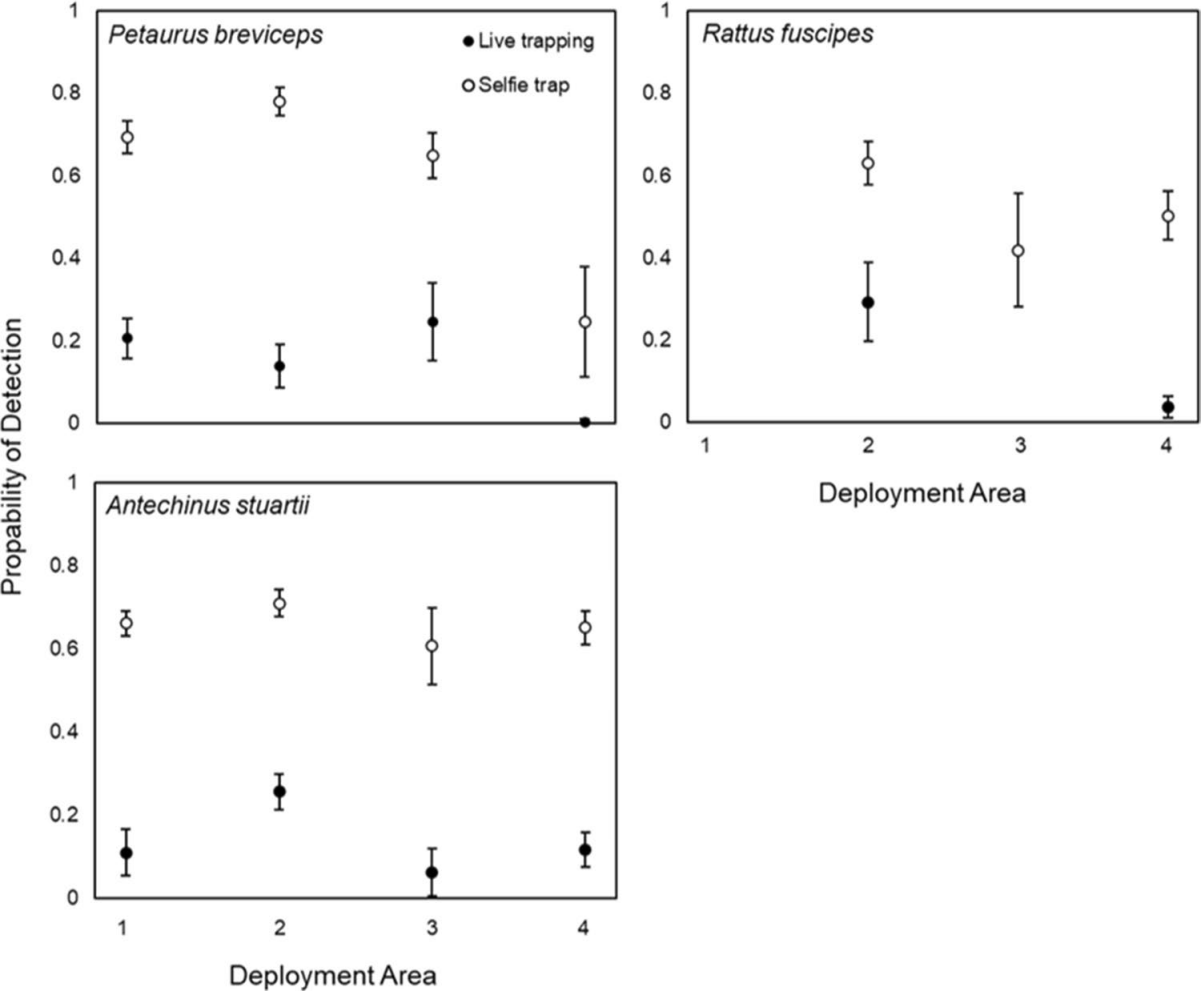
Relationship between detection probabilities estimated by single-season occupancy models in each deployment area and detection methods (live trapping vs. selfie traps), for the three small mammal species captured in the study. Each method used a 7-night dataset for comparative purposes. No captures of *R fuscipes* occurred for area one, and for the live trapping week in area three

Live trapping was implemented across four different areas, for a total of 1,148 trap-nights (Table 1). A total of 104 animals were captured in live traps (Table 1). The live trap success rate of number of individuals per trap night, across all sampled areas, was 3.2% for sugar glider, 5.3% for brown antechinus and 0.6% for bush rat. Probabilities of detection using live trapping was lower than camera trapping, across all species (Fig. 4).

**Table 1:** Summary of live trapping captures of brown antechinus (*Antechinus stuartii*), sugar gliders (*Petaurus breviceps*) and bush rats (*Rattus fuscipes*), for four deployment areas, between August and November 2019

Area	Species	Total no. of individuals	Total no. of captures
Area 1	*Brown antechinus*	10	11
	*Sugar glider*	19	27
	*Bush rat*	0	0
Area 2	*Brown antechinus*	30	41
	*Sugar glider*	11	14
	*Bush rat*	6	9
Area 3	*Brown antechinus*	4	6
	*Sugar glider*	6	8
	*Bush rat*	0	0
Area 4	*Brown antechinus*	17	21
	*Sugar glider*	1	1
	*Bush rat*	1	1

Brown antechinus was caught most often of all three species, with a detection probability ranging from 6.2% to 25.6%. The second highest capture rate was for sugar glider with detection probability ranging from 0.4% to 24.6%. Of all three species, bush rats (the least arboreal) had the largest variation in probability of detection (ranging from no detections to 29.3%).

**Fig. 5:**
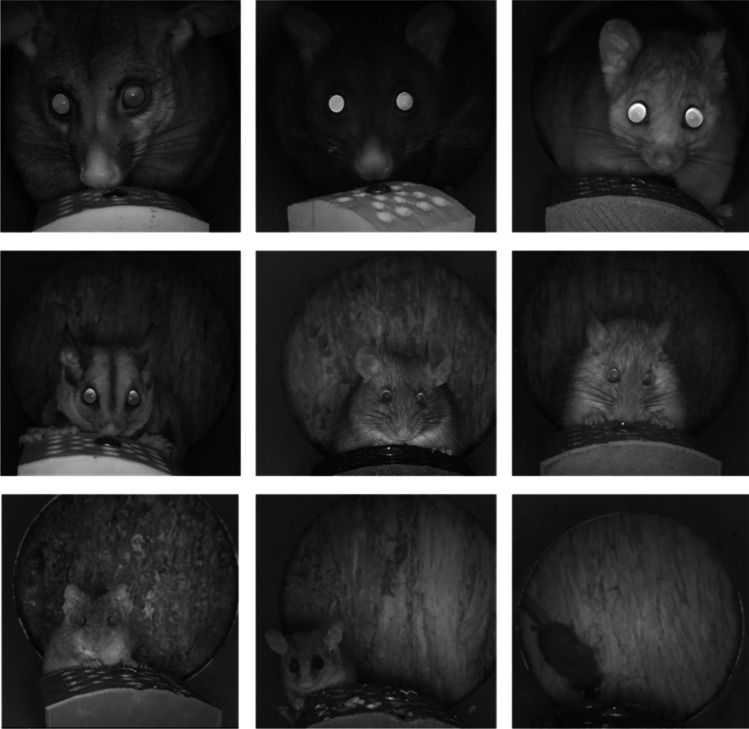
Cropped images from videos documenting all nine mammal species recorded on selfie trap cameras in the study, from top left to bottom right: common brushtail possum (*Trichosurus vulpecula*), southern bobuck (*Trichosurus cunninghami*), common ringtail possum (*Pseudocheirus peregrinus*), sugar glider (*Petaurus breviceps*), bush rat (*Rattus fuscipes*), brown rat (*Rattus norvigecus*), brown antechinus (*Antechinus stuartii*), eastern pygmy possum (*Cercartetus nanus*) and feathertail glider (*Acrobates pygmaeus*)

The total number of camera trap nights across all four areas was 4,592. In total, 76,670 videos were collected (5.6% false triggers). Regarding individual identification of sugar gliders, 41% of all sugar glider videos collected were assigned as “unknown” (14,136) and the remainder identifiable to the individual level. Using the least common interval setting applied (10-min delay), the total number of videos in the final dataset was 28,566, recording nine mammal species (Table 2; Fig. 5).

**Table 2:** Summary of the number of videos recorded for each species (at 10-min intervals), across four deployment areas, between August and November 2019. Unique individuals were identified on cameras footage either unique facial markings, scars or marked ears from live trapping

Area	Species	Number of individuals identified	Total no. of videos
Area 1	*Antechinus stuartii*	30	4959
	*Petaurus breviceps*	61	1859
	*Pseudocheirus peregrinus*	1	1
	*Rattus fuscipes*	2	39
	*Rattus norvegicus*	8	366
	*Trichosurus vulpecula*	4	35
Area 2	*Antechinus stuartii*	26	3789
	*Petaurus breviceps*	83	8172
	*Pseudocheirus peregrinus*	3	9
	*Rattus fuscipes*	11	888
	*Rattus norvegicus*	1	31
	*Trichosurus vulpecula*	1	46
Area 3	*Antechinus stuartii*	3	320
	*Petaurus breviceps*	32	1602
	*Pseudocheirus peregrinus*	1	15
	*Rattus fuscipes*	1	298
	*Rattus norvegicus*	9	1017
	*Trichosurus cunninghami*	1	76
	*Trichosurus vulpecula*	1	67
Area 4	*Acrobates pygmaeus*	1	1
	*Antechinus stuartii*	18	3331
	*Cercartetus nanus*	1	14
	*Petaurus breviceps*	5	63
	*Pseudocheirus peregrinus*	1	1
	*Rattus fuscipes*	3	680
	*Rattus norvegicus*	7	609
	*Trichosurus cunninghami*	1	45
	*Trichosurus vulpecula*	5	233

The camera trap success rate of number of individuals detected per trap night, across all areas, was 3.9% for sugar glider, 1.7% for brown antechinus and 0.4% for bush rat. For all three small mammal species, selfie trap detection probabilities were higher than live trapping detection probabilities (Fig. 4). Detection probabilities for a species using selfie traps ranged from 24.6% to 77.9% for sugar gliders, 60.6% to 70.9% for brown antechinus and no detections to 63% for bush rats.

### Does the duration and trigger interval of selfie trap surveys influence detection rates of individuals and species?

**Fig. 6:**
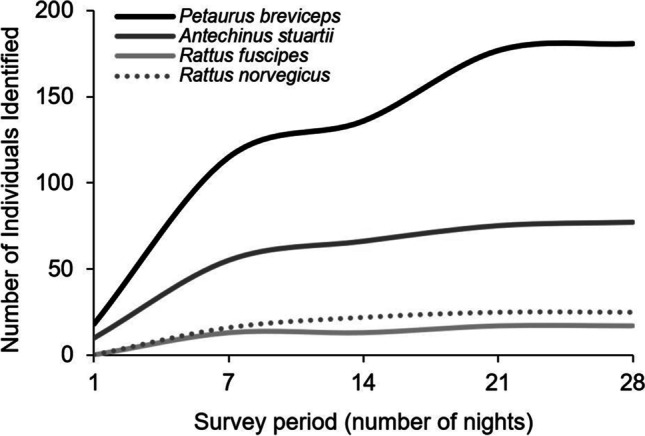
The effect of sampling period on the number of unique individuals identified on selfie traps, for four small mammal species, across all areas between August and November 2019

The detection of unique individuals on selfie traps was impacted by sampling periods (Fig. 6). For the species readily identified through unique facial markings, sugar gliders were most sensitive to survey period, with only 136 individuals identified on selfie traps in the 7-day subset of the data compared to 181 individuals using the full 28-day dataset (Fig. 6).

**Fig. 7:**
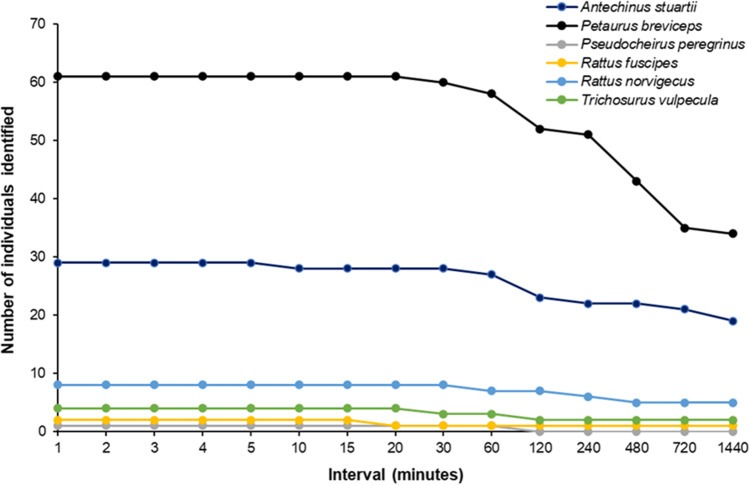
The effect of camera trigger intervals on the number of individuals for six species observed on camera in area one (*n* = 45 selfie traps). The x-axis is not to scale

The dataset from area one provided a unique opportunity to compare the effect of trigger interval, ranging from a one-minute delay to a 24 h delay, on the number of individuals observed on camera (Fig. 7). A delay of up to 20 min did not affect the number of individual sugar gliders detected on camera, whereas a drop in individuals was observed for brown antechinus after a five-minute interval setting was tested.

### Can small mammal abundance be estimated using capture rates from selfie traps?

Of the 16 EUs, two outliers were removed from analyses for sugar gliders as these sites had several cameras positioned on trees with resident sugar glider dens (over inflating the number of videos recorded). Due to varying live trapping rates affecting the number of individual brown antechinus identifiable on camera (one EU relied solely on unique natural markings for individual identification), this outlier was removed as identified through Mahalanobis Distances.

**Fig. 8:**
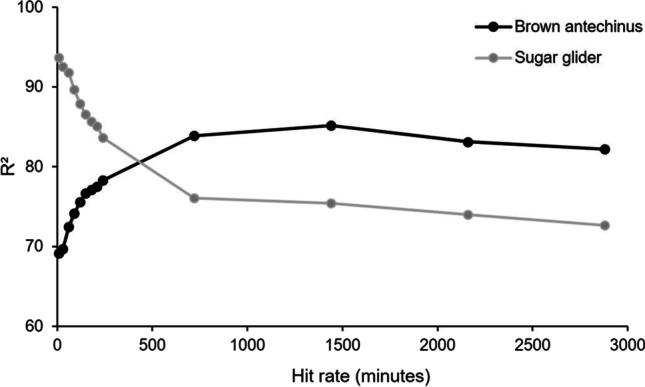
R^2^ values for linear regressions between abundance (number of unique individuals identified on camera) and hit rates (interval between videos recorded) for sugar gliders (*Petaurus breviceps*) and brown antechinus (*Antechinus stuartii*). The hit rate intervals tested were 10, 30, 60, 90, 120, 150, 180, 210, 240, 720, 1440, 2160 and 2880 min

**Fig. 9:**
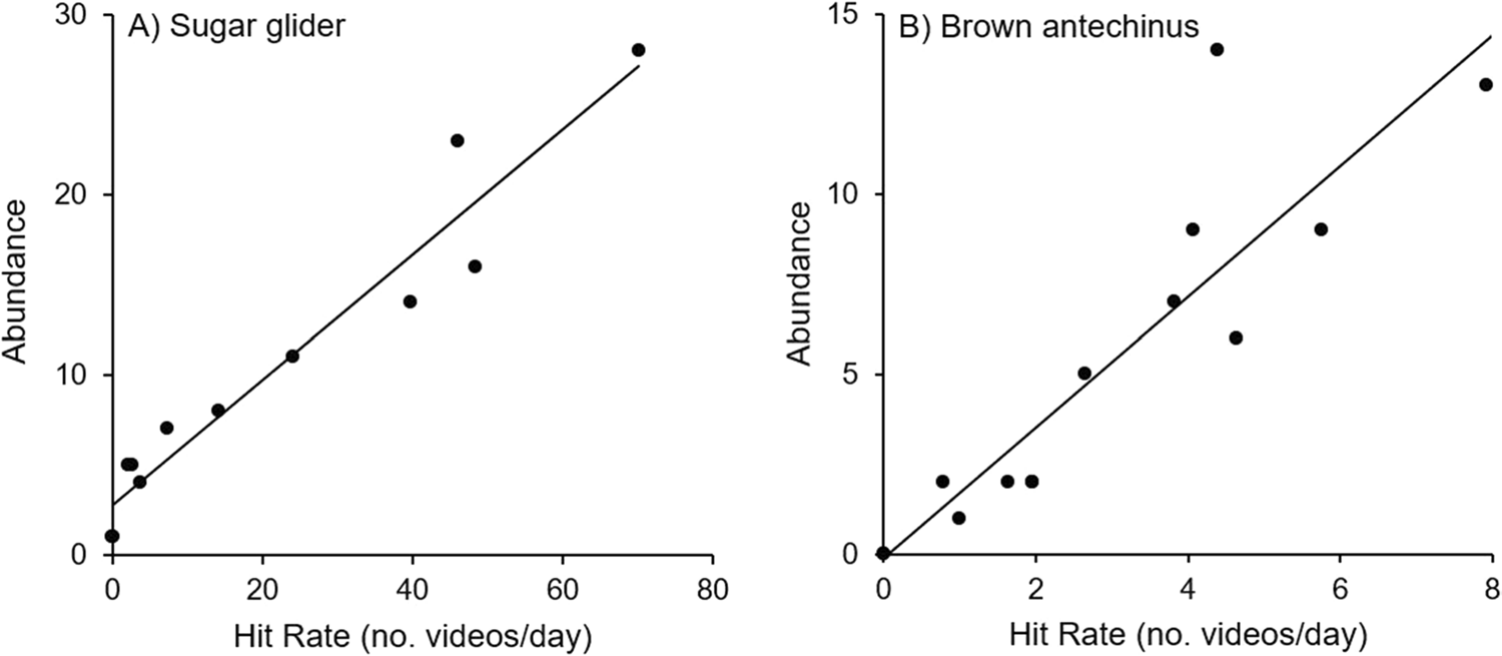
Relationship between hit rates and abundance for A) Sugar gliders (*Petaurus breviceps*) and B) brown antechinus (*Antechinus stuartii*). Hit rates were calculated using a 10-min interval for sugar gliders (R^2^ = 94%), and a 1440-min interval for antechinus (R^2^ = 85%). Abundance was measured as the number of distinct individuals identified on cameras including individuals captured during live trapping

For sugar gliders, the various hit rate intervals applied influenced the goodness of fit, as R^2^ values ranged from 0.72 to 0.94 (Fig. 8). The highest R^2^ value was for the regression between daily average hit rate using a 10-min interval, and this steadily declined as the hit rate interval increased. The opposite was found for brown antechinus, as the regression with the hit window of 1440 min performed best (R^2^ = 0.85) (Fig. 9).

**Fig. 10:**
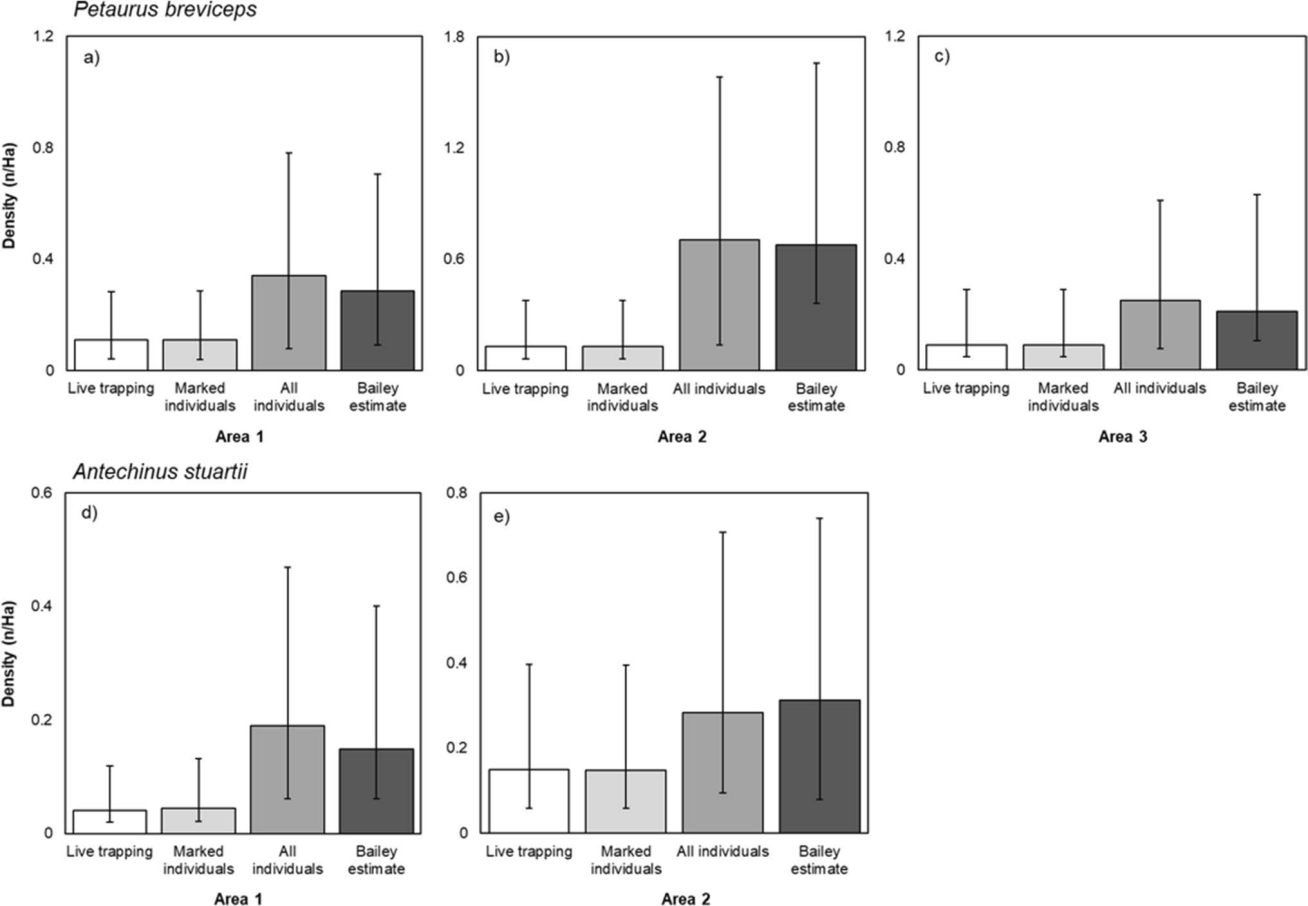
Comparison of density estimates from spatially-explicit capture recapture analyses for *Petaurus breviceps* (**a**–**c**) and *Antechinus stuartii* (**d**–**e**), using the following datasets for each area: simulated live trapping (only the first individual of each species caught on a selfie trap each night was recorded as an observation), marked individuals on camera (using only individuals that had been physically captured and given a permanent unique ear identifier), and all individuals on camera (marked and other unique individuals identified through camera data). The Peterson method (using the Bailey estimate) was calculated using a single marking event (number of individuals physically caught and marked over the course of one week) and a single recapture event (number of individuals seen again on camera over one month). Error bars represent 95% confidence intervals

The two best regression models selected were significant for sugar gliders (F_1,11_ = 147.82, *p* < 0.0001, Fig. 10, Table 3) and brown antechinus (F_1, 12_ = 47.87, *p* < 0.0001, Fig. 9, Table 3).

**Table 3:** Linear regression to predict abundance estimates (number of unique individuals identified on selfie traps) from camera trapping hit rates (hits/day) for sugar gliders (*Petaurus breviceps*) and brown antechinus (*Antechinus stuartii*). Hit rates were calculated using a 10-min interval for sugar gliders, and a 1440-min interval for brown antechinus

Species	Regression equation	Sample size	Mean squared error	Slope standard error	R^2^
*Petaurus breviceps*	Abundance = 2.06 + 0.36*Hit rate	14	2.285	0.027	94%
*Antechinus stuartii*	Abundance = -0.126 + 1.81*Hit rate	15	2.066	0.210	85%

### Can small mammals be individually identified using the selfie trap in order to estimate density at a site?

Across all areas, density estimates from the four density calculations ranged from 0.09/ha to 0.70/ha for sugar glider and 0.04/ha to 0.31/ha for brown antechinus (Fig. 10). For both species, using live trapping data or only physically marked individuals on camera, resulted in underestimation of density compared to using the method of selfie traps that observed greater numbers of individuals in total (Fig. 10).

## Discussion

### Is the probability of detection of small mammals higher for selfie traps when compared to live trapping?

Our data demonstrate that selfie traps are effective in distinguishing morphologically similar small mammals. When comparing the probability of detecting the presence of the three small mammal species (sugar gliders, brown antechinus, and bush rats) in the study area, selfie traps were superior to Elliott traps across a comparative 7-day selfie trap effort. Other studies have found camera traps to be more effective at detecting small mammal species compared to live trapping (De Bondi et al. 2010; Greene et al. 2016; Thomas et al. 2020). As cameras are an “open” trap throughout the night, it is likely the most important factor that makes camera trapping a highly efficient method for detecting multiple species. In the case of the selfie trap, the enclosed space provides shelter and protection which encourages extended and repeated visits by animals.

### Does the duration and trigger interval of selfie trap surveys influence detection rates of individuals and species?

The effect of different sampling intervals on the number of individuals identified on camera was most pronounced for sugar gliders. This is because the species was the most readily distinguishable at the individual level. However, many videos had to be assigned as “unknown” due to similarities, unkept fur, wet fur, or no face was visible in the footage. To increase precision of individual identities within datasets, initial live trapping to mark individuals is recommended where possible. Our results suggest that a minimum of 28 days was required to effectively sample enough individuals, however, to ensure enough recaptures, we recommend 56 days as a minimum. Research from forests in Tasmania found that two months were needed when using selfie traps to obtain enough captures and enough footage overall for identification of recaptures (pers. comm. G. Owens). This also enhances the ability of the selfie trap to effectively record species richness (Gracanin and Mikac 2022). In addition, when considering what motion delay should be programmed into the camera, we argue that an interval of 10 min is able to maintain a balance between obtaining enough videos for individual identification and generating a manageable quantity of data. Only one other study has investigated the effect of motion sensor trigger intervals on detection probability and occupancy of species (Lepard et al. 2019); they found a similar result, where increasing the delay (intervals ranging from 10 s to 10 min) had low impact on detection probability; however, intervals ranging from 10 to 60 min had much larger impacts on detection probabilities. The same authors were unable to investigate the effect of trigger delays on abundance and density estimates; however, our study suggests substantial decreases in abundance estimates after 60 min for sugar glider and brown antechinus. The decrease in the number of individuals identified on selfie trap footage however are only predictive, as we are unable to account for whether a video file included in the various interval datasets displayed enough detail for confident identification. Thus, our results may not reflect the true sensitivity to trigger delays, though a delay of ten minutes is shown to detect enough species and individuals, and reduce data management and processing fatigue (Lepard et al. 2019).

### Can small mammal abundance be estimated using capture rates from selfie traps?

Our study is the first to estimate abundance and density of sugar gliders, brown antechinus and bush rats using camera traps. The data indicate that rates of camera footage (hit rates) are an accurate method for estimating abundance, and thereafter density. This demonstrates that the use of the selfie trap is a viable alternative to live trapping small mammal species. However, as brown antechinus and bush rats are more ground-dwelling, the results presented here likely reflect a reduced rate of capture as selfie traps and live traps were positioned in trees. Further experimentation using ground-placed selfie traps confirm whether the relationship found between brown antechinus abundance and rates of camera footage is repeatable elsewhere and identify other potential trends in ground-dwelling small mammals.

The effect of various hit rate intervals to calculate rates of footage and their relationship with abundance varied for brown antechinus and sugar gliders. The correlation between hit rate and abundance was sensitive to hit rate intervals. This is likely due to the variation in the ability of the user to identify antechinus individuals, compared to sugar glider that have unique head stripe patterns and are overall larger, allowing for clearer views of natural scars on the ears. Future investigation into hit rates recorded at intervals lower than 10 min would be of value, as these could increase the precision (Villette et al. 2017). The sensitivity to hit rate intervals also reflects heterogeneity in the amount of time individuals spend inside the selfie traps. Where an individual spends more time inside the selfie trap than others, the number of videos recorded is not only due to the population density but variation in individual species behavior. Another factor that likely contributes to video footage capture rates is that some species dominate and exclude others. For example, brown rats (*Rattus norvigecus*) were observed to chase sugar gliders and vice versa. In the case of brown antechinus, as they are substantially smaller, this species was always observed fleeing if any other species was present. The presence of scats, urine and scent marking could have also affected the visitation rates of different species. This however was only an issue where brown rats left feces, though it was still observed that many other species still visited.

### Can small mammals be individually identified using the selfie trap in order to estimate density at a site?

The variation in SECR density estimates for brown antechinus and sugar glider from datasets representing different collection methods reinforces the idea that selfie traps are more accurate. This is simply due to the distinct capability of selfie traps to record more individuals than live trapping. Our density estimates for sugar gliders using the selfie trap is within the range of other studies investigating glider density, relative for each season surveyed (Quin 1995; Jackson 2000). However, our SECR results sit mostly in the lower estimates and this is likely due to the highly fragmented habitat surveyed, as well as SECR analyses can often result in large confidence intervals as most studies are rarely able to achieve ideal maximum recapture rates (Gray and Prum, 2012; McGregor et al., 2015; Mohamed et al., 2021). Thus, our final recommendations are to utilize a minimum of eight weeks for capture-recapture analyses to increase precision of density estimates.

### Is camera trapping using the selfie trap a more cost-effective method than standard live trapping?

Depending on the purpose of survey work, live trapping is a less expensive method for presence/absence surveys (Table 4). However, compared to selfie traps, live trapping had very low probability of detection for this study’s small mammal species, thus making selfie traps the better choice. Despite the high initial upfront cost, its use over longer survey periods (or simply its repeated use over the long term) results in an overall cost-effective method compared to live trapping (Table 4). The selfie trap method is also important for when considering time requirements, as live trapping requires more staff time.

**Table 4:** Comparison of the estimated costs of camera trapping using the selfie trap method and live trapping, when considering a one, four- and eight-week surveys, under this study’s ecological conditions for one area. Hourly rates of pay were applied using relevant university casual rates, for two fieldwork team leaders. The number of hours taken to sort footage (to species) is assumed to be undertaken by an experienced and trained individual

Method	Expense item	Unit value	One-week survey quantity	Total	Four-week survey quantity	Total	Eight-week survey effort quantity	Eight-week survey effort quantity	Total
Selfie trap	Selfie trap	$338.56	50	$16,928.00	50	$16,928.00	50	50	$16,928.00
	Batteries	$0.30	400	$120.00	400	$120.00	800	800	$240.00
	Fuel	$1.50	30L	$45.00	45L	$67.50	75L	75L	$112.50
	Bait	$50	1	$50.00	2	$100.00	4	4	$200.00
	Fieldwork salary	$56.75	28 h	$1,589.00	42 h	$2,383.50	70 h	70 h	$3,972.50
	Sorting of footage	$56.75	25	$1,418.75	50	$2,837.50	100	100	$5,675.00
			Grand total:	$20,150.75	Grand total:	$22,436.50	Grand total:	Grand total:	$27,128.00
Live trapping	Accommodation	$231.50	7 Nights	$1,620.50	28 nights	$6,482.00	56 nights	56 nights	$12,964.00
	Fuel	$1.50	56L	$84.00	224L	$336.00	448L	448L	$672.00
	Bait	$50	1	$50.00	4	$200.00	8	8	$400.00
	Traps	$40	50	$2,000	50	$2,000	50	50	$2,000
	Fieldwork salary	$56.75	56 h	$3,178.00	224 h	$12,712.00	448 h	448 h	$25,424.00
			Grand total:	$6,932.50	Grand total:	$21,730.00	Grand total:	Grand total:	$41,460.00

Values in $AUD.

### Recommended settings and procedures

In terms of the camera setting of a delay interval, applying a delay of five minutes was ideal for all species in this study based on the subset of data from area one. However, this led to very large datasets, thus a delay of 10 min is recommended. A summary of recommended settings and sampling procedures is provided (Table 5). For studies investigating density and life history of individuals, we strongly recommend performing live trapping where possible to create permanent unique ear notches on small mammals. Our study found that 78% of all sugar glider individuals profiled, relied on ear scars, ear markings and/or tail tip color, for confident assignment as an individual. The remainder had head stripe patterns alone to identify individuals as unique, though this method meant stricter protocols for assignment (e.g., anything with unkept fur had to be assigned as “unknown”). In the pilot study by Gracanin et al. (2018), the authors predicted that unique facial markings (i.e., head stripes for sugar gliders) could be used for individual identification; however, this study found that permanent markers (either ear notching or natural ear scars) were more effective. Thus, the selfie trap provides clear, sharp resolution of animal ears for individual identification purposes, even for very small mammals such as *Antechinus sp.* (20 g).

**Table 5:** Final recommendations for use of selfie traps for small to medium mammal species survey and density estimation

Camera trapping facet	Recommendations
Interval between triggers	10-min interval
Period for general survey work	Minimum 56 days
Period for population estimates	Minimum 56 days
Placement	Arboreal and ground (dependent on target species)
Rebaiting frequency	Every ten days
Tagged ears?	When possible, perform tagging of ears to increase user’s ability to distinguish between individuals of small mammals

## Conclusions

As detection methods develop over time, researchers are provided with many opportunities for gathering ecological and biological data. Information from these methods can result in changes in conservation policy and management, thus methods and survey designs must be relevant to effectively collect data (Clare et al. 2017). Our findings demonstrate the utility of selfie traps when compared to the traditional method of live trapping for detecting and surveying for small mammals.

The application of the selfie trap is not limited to the species within this study’s system, as there are thousands of other small mammal species worldwide. For example, it may have applications for squirrel, rat, mice, vole, lemming, weasel, stoat and gopher species, as well as small species of arboreal monkeys and possums. Selfie traps are capable of distinguishing between many similar appearing species (e.g., *Rattus fuscipes, Rattus rattus, Rattus norvigecus* and *Petaurus breviceps*, *Petaurus norfolcensis*) which allow for accurate species presence and absence surveys, without the need for live trapping.

## Supplementary Information

Below is the link to the electronic supplementary material.

Supplementary file1 (PDF 227 KB)

## Data Availability

The datasets generated during the current study are available from the corresponding authors on reasonable request.
