# Supplementary material for: Estimating the density of small mammals using the selfie trap is an effective camera trapping method
Source: Mamm Res. 2022 Jul 22;67(4):467–82. doi: 10.1007/s13364-022-00643-5 (PMC9304545; doi:10.1007/s13364-022-00643-5)
Supplement: Supplementary file 1 — Supplementary file1 (PDF 227 KB) [file 13364_2022_643_MOESM1_ESM.pdf]

Appendix 1: Procedure used to ensure accurate identification of individuals, and test of procedure by four observers.

After videos have been sorted to species, the observer can begin investigating the sugar glider footage:

1. Begin viewing videos in a sequential order, paying attention to date and time.

2. Identify potential visits (series of videos) and sort them in folders if needed (‘Visit 1’, ‘Visit 2’, ‘Visit 3’), as well as sorting them as males where possible (prominent bald patch).

3. Any videos with no clear ear features or clear face shots present, place immediately into “unknown” folder.

4. Where a clear face and ears is visible in a single video, take a screenshot (to begin creating profiles where needed; in Microsoft Word, Excel or PowerPoint).

5. Continue observing and identifying clear faces and/or ears.

**Example of a clear identifiable face:**	**Example of a face that cannot be used:**
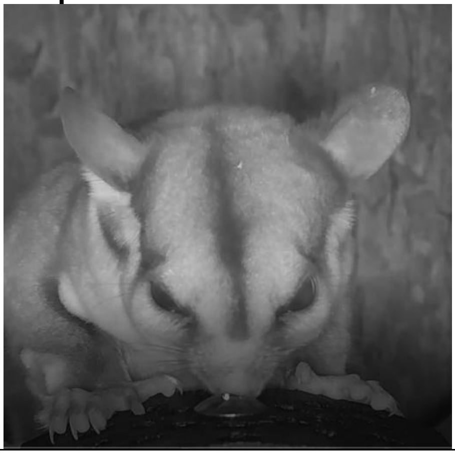	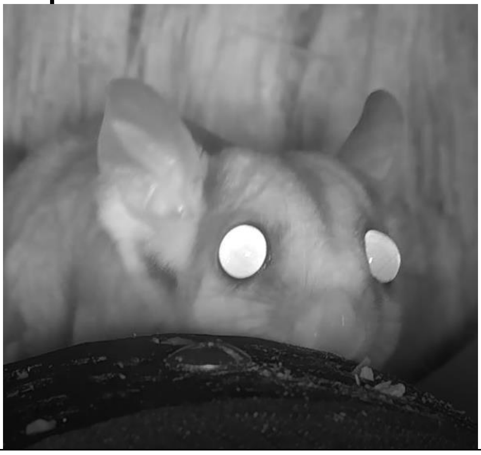

6. Any video that cannot be used (e.g., back turned to camera, captures glider leaving etc) is placed into an “unknown individual” folders.

**Examples of videos that are assigned as “unknowns”:**	
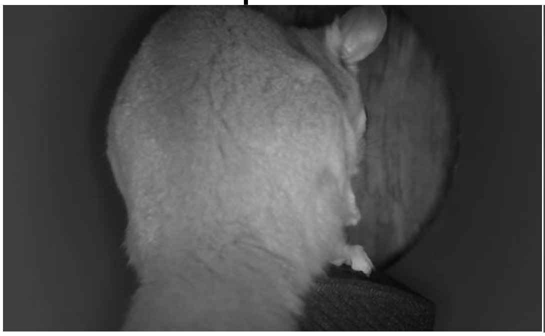	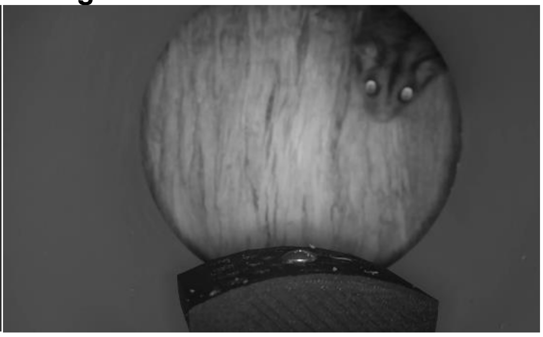

7. Once an initial view of the footage has been performed, begin to compare face shots collected so far.

8. Create profiles for easily identifiable individuals.

Examples of easily identifiable individuals:

**Example 1** 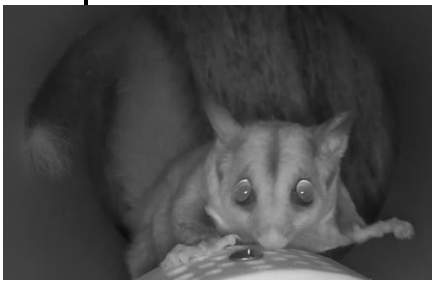 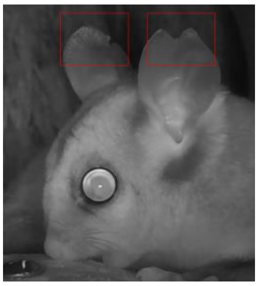
**Example 2** 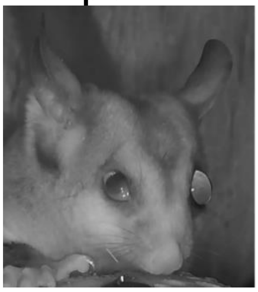 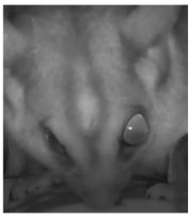
**Example 3** 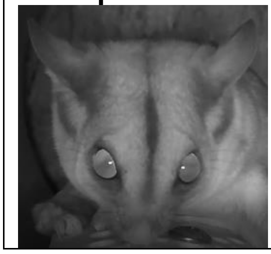

9. Re-watch the footage, assigning videos to individuals identified.

10. For individuals solely reliant on facial markings, fur must not be wet or unkept.

11. Whenever there is uncertainty for an individual to be assigned, assign it to “unknown individual”.

12. The use of tail tip colour and spatial location can be used to assist with distinguishing individuals.

13. If two or more individuals are seen in the one video, make copies of each file and assign each individual accordingly (only if they can confidently identified within the one video).

Testing identification method

To confirm accuracy of this procedure, three observers were given a sub-sample of the data from four sites and tasked with identifying unique individual gliders (Table 1). All observers were able to identify the same number of distinct individuals, except for observer three whom missed individual 12 (Table 1).

Table 1. Number of videos assigned to individual sugar gliders and those classed as "unknowns" by four observers, for a sub-sample of the data (N = 1,336).

Site	Video assignment	Sex	Identifying features	Original observer (A.G)	Observer 1	Observer 2	Observer 3
A1_T17	Individual 1	F	Ear marked	271	265	251	240
A1_T17	Individual 2	F	Natural ear scar	86	83	65	70
A1_T17	Individual 3	F	Natural ear scar	2	1	1	1
A1_T17	Individual 4	M	Natural ear scar	144	185	131	136
A1_T17	Unknowns	-	-	521	490	576	577
A2_T43	Individual 5	M	Head stripe	22	20	18	19
A2_T43	Individual 6	F	Natural ear scar	52	49	44	41
A2_T43	Unknowns	-	-	61	66	73	75
A3_T05	Individual 7	F	Ear marked	30	32	30	30
A3_T05	Individual 8	M	Ear marked	2	2	2	1
A3_T05	Individual 9	M	Natural ear scar	33	35	33	30
A3_T05	Unknowns	-	-	60	56	60	64
A4_T36	Individual 10	F	Ear marked	12	15	12	12
A4_T36	Individual 11	F	Ear marked	8	8	8	7
A4_T36	Individual 12	M	Stripe & white-tip tail	2	2	2	0
A4_T36	Individual 13	M	Natural ear scar	1	1	1	1
A4_T36	Unknowns	-	-	29	26	29	32
